# Molecular prevalence and phylogenetic analysis of hemotropic *Mycoplasma* species in cats in different regions of Iran

**DOI:** 10.1186/s12866-024-03356-8

**Published:** 2024-06-08

**Authors:** Elham Hoseinpoor, Ali Goudarztalejerdi, Alireza Sazmand

**Affiliations:** Department of Pathobiology, Faculty of Veterinary Medicine, Bu-Ali Sina University, Hamedan, 6517658978 Iran

**Keywords:** Emerging pathogens, Hemotropic mycoplasmas, *One Health*, PCR, Zoonoses

## Abstract

**Background:**

Hemotropic *Mycoplasma* species (hemoplasmas) cause hemolytic anemia in cats worldwide and are recognized as emerging zoonotic pathogens. There is no comprehensive study on the prevalence and species diversity of hemoplasmas in domestic cat populations in different regions in Iran. Thus, the aims of the present study were to provide data on the prevalence and molecular characterization of hemotropic *Mycoplasma* species in apparently healthy cats from six Iranian provinces with different climates. In addition, potential risk factors associated with hemoplasmosis in cats were assessed.

**Results:**

*Mycoplasma* spp. DNA was detected in the blood of 56 / 361 cats (15.5%) using genus-specific PCR. Further examinations with species-specific PCR and Sanger sequencing showed that 38 cats (10.5%) tested positive for *Candidatus* Mycoplasma haemominutum (CMhm), 8 cats (2.2%) tested positive for *Mycoplasma haemofelis* (Mhf), and 2 cats (0.6%) tested positive for *Candidatus* Mycoplasma turicensis (CMt). Co-infection with CMhm, and Mhf was observed in 7 cats (1.9%). One cat (0.3%) showed mixed infection with CMhm, Mhf, and CMt. There were statistically significant relationships between *Mycoplasma* positivity and being female, living in shelter (cattery), and being over 3 years old (*P* < 0.05). No significant association was observed for the cat breed and sampling localities.

**Conclusions:**

Current study findings revealed that hemoplasma infections are common among Iran cat populations. Considering the impact of such emerging zoonotic pathogens on the *One Health*, routine screenings, increasing public awareness, effective control, and prophylactic strategies for minimizing infection in cats and subsequently in human are strongly recommended.

## Background

Recently, the zoonoses and emerging pathogens have received increasing attention in public and animal health [1, 2]. Hence, there is a need for current epidemiological data to plan for rapid identification and implementation of effective control strategies to decrease their incidence in animals and humans [3]. Among domestic animals around us, cats are known to be important sources for human infection by various zoonotic pathogens [1, 4].

Feline vector-borne diseases which are transmitted by a variety of vectors, such as ticks, fleas, mosquitoes, and phlebotomine sand flies are of increasing importance in both public and animal health worldwide [1, 2]. It is partly because of their increasing incidence, challenges in their diagnosis (e.g. long incubation periods, no pathognomonic clinical signs) and subsequently their control [5]. Even if some cat habits such as self-grooming can potentially minimize the establishment of some ectoparasites e.g. ticks, their outdoor access exposes them to infected arthropod vectors and consequently to the pathogens [6]. Furthermore, vertical transmission of some pathogens from persistently infected queens makes the situation even more complex especially in regards to the control of zoonotic pathogens [7].

Among vector-borne pathogens, hemotropic *Mycoplasma*s (hMycos) are ubiquitous [8]. These pathogens are small, gram-negative, uncultivable, pleomorphic, and cell-wall-free bacteria which parasitize red blood cells [9–11]. They are known to cause infections ranging from asymptomatic to acute infectious anemia in a wide variety of domestic and wild animals, including cats [10–12]. Importantly, some hMycos are capable of infecting humans [13–17].

In cats, hMycos cause clinical features include lethargy, anorexia, fever, and hemolytic anemia [10]. Three hMycos namely *Mycoplasma haemofelis* (Mhf), *Candidatus* Mycoplasma haemominutum (CMhm), and *Candidatus* Mycoplasma turicensis (CMt) are known to infect cats [9, 11, 18]. However, they have different pathogenicities for instance Mhf causes severe and fatal hemolytic anemia, CMhm causes only minimal clinical signs and anemia is not usually induce, and CMt can induce mild to moderate anemia in infected animals [9, 10, 18, 19]. Currenlty, transmission routes of hMycos remain largely unknown but the bite of fleas, through the saliva and fecal material, and aggressive interaction between cats have been suggested [20–22].

Previous studies reported prevalence of cat hemoplasmosis in different regions of the world up to 54.3% in South Korea [23]. In the Middle East countries feline hMycos have been reported from Türkiye [24, 25], Egypt [26], Qatar [27], and Saudi Arabia [28]. In Iran however, few studies with limited number of animals from Tehran (185 domestic cats), Kerman (142 stray cats), Tehran (52 stray cats), and Tehran (19 wild felids) have been conducted [29–32]. Therefore, the purpose of the current study was to provide a comprehensive information about the prevalence and molecular characterization of hMyco in apparently healthy cats from six Iranian provinces with different climates. In addition, potential risk factors associated with hemoplasma infections in cats were assessed.

## Results

### Molecular prevalence

**Fig. 1:**
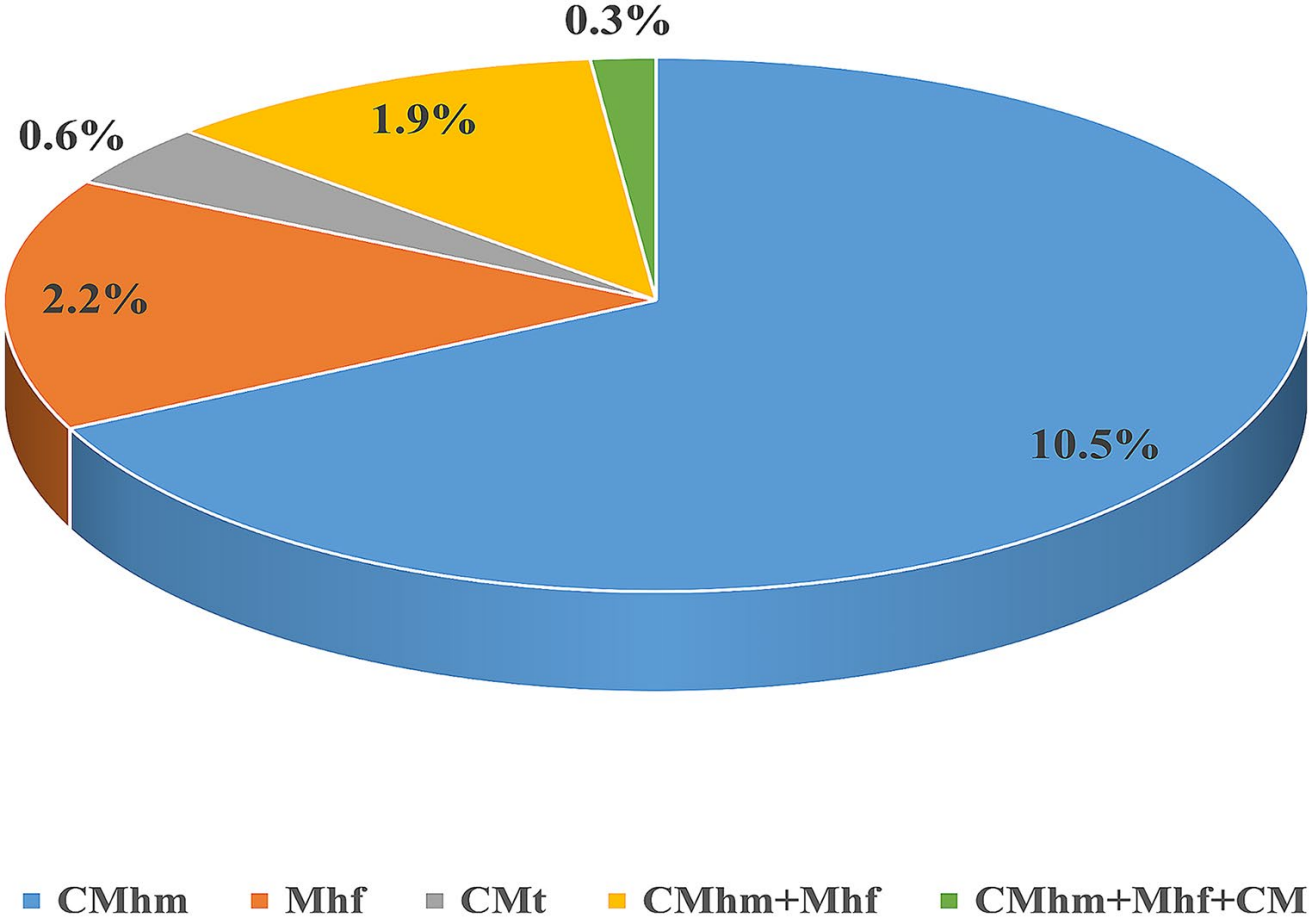
Molecular prevalence of hemotropic *Mycoplasma* spp. in 361 apparently healthy cats from six Iranian provinces. CMhm: *Candidatus* Mycoplasma haemominutum, Mhf: *Mycoplasma haemofelis*, CMt: *Candidatus* Mycoplasma turicensis

DNA of hMycos was detected in 56 / 361 cats (15.5%, 95% CI = 11.6–19.1) using genus-specific PCR. Further species-specific PCRs and Sanger sequencing revealed positivity of 38 cats for CMhm (10.5%, 95% CI = 7.5–13.9), eight for Mhf (2.2%, 95% CI = 0.8–3.9), and two for CMt (0.6%, 95% CI = 0–1.4). Co-infection with CMhm and Mhf was observed in seven cats (1.9%, 95% CI = 0.6–3.6). One cat (0.3%, CI = 0–0.8%) was positive for all three CMhm, Mhf, and CMt (Fig. 1).

### Sequencing and phylogenetic analysis

BLAST analysis of six CMhm nucleotide sequences obtained in this study showed > 99% similarity with previously GenBank entries for cat (*Felis catus*) from Iran (HE804776, MW555992), Persian leopard (*Panthera pardus*) from Iran (KU852586, KU85258), European wildcat (*Felis silvestris*) from Germany (ON202711), also domestic cat from Chile (MN543623) and South Korea (KY432679). Nucleotide sequences of Mhf isolated from a cat of Kermanshah showed 99% similarity with the bacteria in cats from Iran (MT428555), Thailand (KJ858515) and Brazil (OQ397119). BLAST analysis also showed > 98.5% identity of CMt isolated from a cat in Hamedan with GenBank deposited entries of cats from South Korea (KY046312), Italy (KR905460) and Taiwan (JQ689950).

**Fig. 2:**
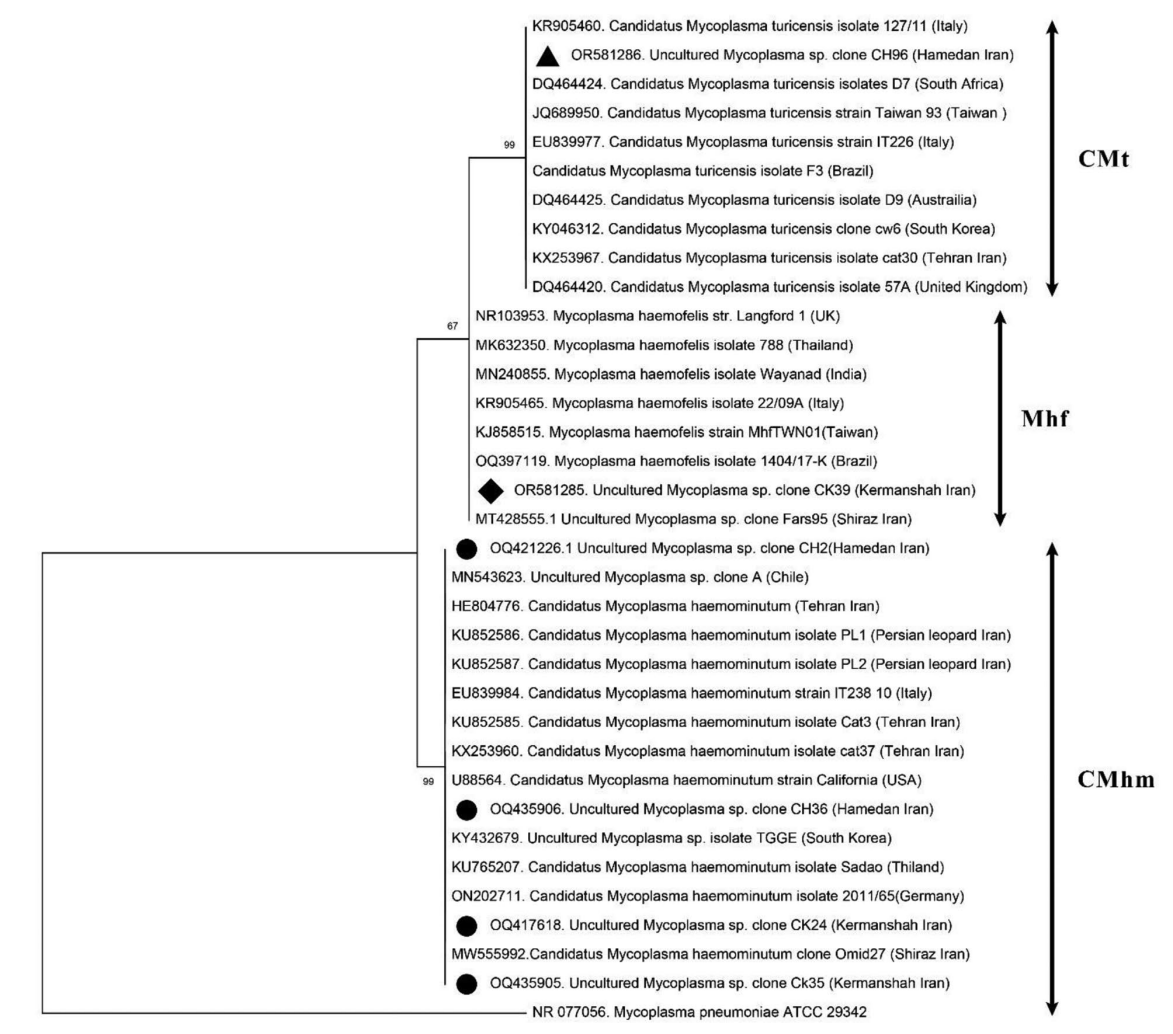
Phylogenetic tree of *Mycoplasma* spp. isolated in this study based on a 595 bp fragment of 16 S rRNA gene using the Maximum Likelihood method. Numbers at branches represent bootstrap support levels (1000 replicates). *Mycoplasma pneumoniae* (GenBank: NR077056) was used as the outgroup. The CMhm, Mhf, and CMt sequences generated in this study are indicated by black circle (●), diamond (♦), and triangle (▲) symbols, respectively

The constructed Maximum Likelihood (ML) tree based on the partial 16 S rRNA gene sequences of *Mycoplamsa* spp. showed that all sequences herein detected, clustered in well-supported clades with other known and previously reported hMycos (Fig. 2). Representative sequences of hMyco detected in this study were deposited in the GenBank^®^ database under the accession numbers OQ421226, OQ435906, OQ417618, OQ435905 for CMhm, OR581285 for Mhf, OR581286 for CMt.

### Risk factors analysis

Statistically significant relationships were observed between PCR positivity of *Mycoplasma* spp. (i.e. genus-species primers) and being female, living in shelter, and being older than 3 years (*P* < 0.05). In addition, positivity was more prevalent in Domestic Shorthair breed (16.55%) and in cats of Khorasan Razavi (25.96%). No significant association were observed in cats for breed and sampling areas (Table 1).

**Table 1:** Prevalence of *Mycoplasma* spp. in 361 cats of Iran according to different variables

		No. PCR positive cats (%)					
Variable	**No.**	**CMhm**	**Mhf**	**CMt**	**CMhm + Mhf**	**CMhm + Mhf + CMt**	**Total**
**Gender**							
Male	135	9 (6.66)	2 (1.48)	0	1(0.74)	0	12 (8.88)
Female	135	19 (14.39)	3 (2.27)	1 (0.75)	3 (2.27)	0	26 (19.69)^*^
No data	91	10 (10.63)	3 (3.19)	1 (1.06)	3 (3.19)	1 (1.06)	18 (19.14)
**Age**							
≤ 1 year	20	1(4.76)	0	0	0	0	1 (4.76)
1–3 year	126	7 (5.60)	1(0.80)	0	2 (1.60)	0	10 (8.00)
≥ 3 years	72	14 (20.58)	3 (4.41)	0	0	0	17 (25.00)^*^
No data	143	16 (10.88)	4 (2.72)	2 (1.36)	5 (3.40)	1 (0.68)	28 (19.04)
**Breed**							
Domestic Shorthair	151	15 (9.93)	3 (1.98)	2 (1.32)	4 (2.64)	1 (0.66)	25 (16.55)
Persian	22	0	0	0	0	0	0
Scottish Fold	28	1 (3.57)	1 (3.57)	0	0	0	2 (7.14)
British Shorthair	4	0	0	0	0	0	0
Other	156	22 (14.10)	4 (2.56)	0	3 (1.92)	0	29 (18.58)
**Living condition**							
Cattery	41	9 (21.95)	2 (4.87)	1 (2.43)	1 (2.43)	1 (2.43)	14 (34.14)^*^
Pet	134	4 (2.98)	0	0	0	0	4 (2.98)
Stray	159	23 (14.46)	5 (3.14)	1(0.62)	6 (3.77)	0	35 (22.01)
No data	27	2 (7.40)	1 (3.70)	0	0 (00)	0	3 (11.11)
**Province**							
Khorasan Razavi	104	20 (19.23)	4 (3.84)	0	3 (2.88)	0	27 (25.96)
Kermanshah	85	11(12.94)	3 (3.52)	0	1 (1.17)	0	15 (17.64)
Hamedan	52	2 (3.84)	1 (1.92)	2 (3.84)	3 (5.76)	1 (1.92)	9 (17.30)
Kerman	46	0	0	0	0	0	0
Tehran	45	2 (4.44)	0	0	0	0	2 (4.44)
Yazd	29	3 (10.34)	0	0	0	0	3 (10.34)

* *P* < 0.5

## Discussion

This first comprehensive epidemiological survey on apparently healthy domestic cats from Iran shows silent circulation of all three feline-infective hemotropic *Mycoplasma* species in different regions of the country implying its neglected health issues for cats and also the public. Infection of 15.5% of examined cats with at least one hemotropic *Mycoplasma* species is lower than previous studies in Iran i.e. 22–32.7% in Tehran [29, 31, 32] and 18.3% in Kerman [33], also reports from Western Asian and North African countries e.g. 19.3% in Türkiye [25], 27.2% in Saudi Arabia [28] and 20% in Egypt [26], but higher that some other reports e.g. 5.9% in Qatar [27] and 11.4% [24] in Türkiye. However, the molecular prevalence of hemoplasmas in cats in this survey was similar to studies from Germany (15.6%) [34], East and Southeast Asia (16.13%) [26], Brazil (14.6%) [35], Scotland (14.3%) [36], and UK (14%) [37]. This difference in prevalence can be due to environmental and climatic factors, veterinary care, cat populations health status and living condition, availability of arthropod vectors, and methodology e.g. conventional *vs*. real-time PCR [12, 22, 36, 38, 39].

Positivity of 38 cats (10.5%) for CMhm, eight cats (2.2%) for Mhf, and two cat (0.6%) for CMt is in agreement with previously published studies reporting CMhm as the most prevalent species followed by Mhf and CMt [18, 25, 39]. In other studies, from this region, in India 6.3%, 1.8% and 0.9% of apparently healthy cats were positive for CMhm, Mhf, and CMt [40], in Saudi Arabia 13.6% of cats were positive for CMhm and 13.6% of cats were positive for Mhf [28], and in Egypt 18% and 2% of cats were positive for CMhm and Mhf [26]. Similarly, in European countries the frequency of CMhm > Mhf > CMt has been repeatedly reported e.g. from Spain (CMhm: 9.9%; Mhf: 3.7%; CMt: 0.5%) [41], Germany (CMhm: 8.9%; Mhf: 7.7%; CMt: 2.2%) [34] and Italy (CMhm: 9.9%; Mhf: 01.5%; CMt: 0.2%) [42]. This common observation has been associated with higher efficiency of CMhm in infection of and multiplication in cats than Mhf and CMt [22]. In contrast, Mhf was reported to be more common than CMhm in one study from Iran which included only clinically ill cats presenting anemia [31] making the finding reasonable as Mhf is the most pathogenic *Mycoplasma* species infecting cats [43, 44].

Co-infection with more than one species was observed in eight cats (2.2%), seven (1.9%) with CMhm + Mhf, and one with CMhm + Mhf + CMt. Similarly, multiple infection with the same two [39, 45] and three [25] pathogens detected in this study were reported suggesting co-transmission of feline hemoplasmas.

In the present study, living conditions, and age were significantly associated with hemoplasma infection. The older cats were infected more often than younger ones which is in line with previous studies [22, 31, 34, 45], and could be due to longer exposure time to pathogen and increasing risk of acquiring chronic subclinical infection over their lifetime [38, 42]. The significantly higher prevalence of hemoplasmas infection in shelter cats in this study is also similar to that reported in previous studies, where it has been showed that shelter animals are at higher risk of hemoplasma due to their behavior (e.g., fighting or biting), higher prevalence of ectoparasitic infestations in shelter cats, and below-standards sanitation of these shelters [5, 12, 46–48]. Finding of more positive females cats however, is in contrast with some studies that reported males are more likely to be infected with hemoplasmas [22, 41, 45, 49] possibly because most of the female cats (32.6%) in this study were older than three years. Furthermore, in the present study, in line with previous investigations reporting that there was no significant association between hemoplasmosis and the breed of cats [7, 12, 50] breed was not a risk factor, although Domestic Shorthair cats displayed a higher infection rate (16.55%) than other breeds which again can be due to their proportion (41.82%) in the study.

The major limitations of the present study were the lack of information about the clinical history of the sampled cats especially antibiotic therapy e.g. with doxycycline and enrofloxacin which are known to control the infection. Furthermore, we could only test low number of pet cats and sequence limited samples because of resources. For future studies, inclusion of hematology and biochemistry profile of cats in data analysis, investigation of feline immunodeficiency virus (FIV) and feline leukemia virus (FeLV) infection status, and application of more sensitive diagnostic methods such as real-time PCR are suggested.

## Conclusion

This first comprehensive molecular prevalence study in domestic cats from Iran showed silent circulation of all three feline-infective hemotropic *Mycoplasma* species in different regions of the country implying its neglected health issues for cats and also the public. Considering the impact of such emerging and zoonotic pathogens on the *One Health*, routine screenings, increasing public awareness, effective control and prophylactic strategies for minimizing infection in cats and subsequently in human are strongly recommended.

## Methods

### Sample collection and study area

**Fig. 3:**
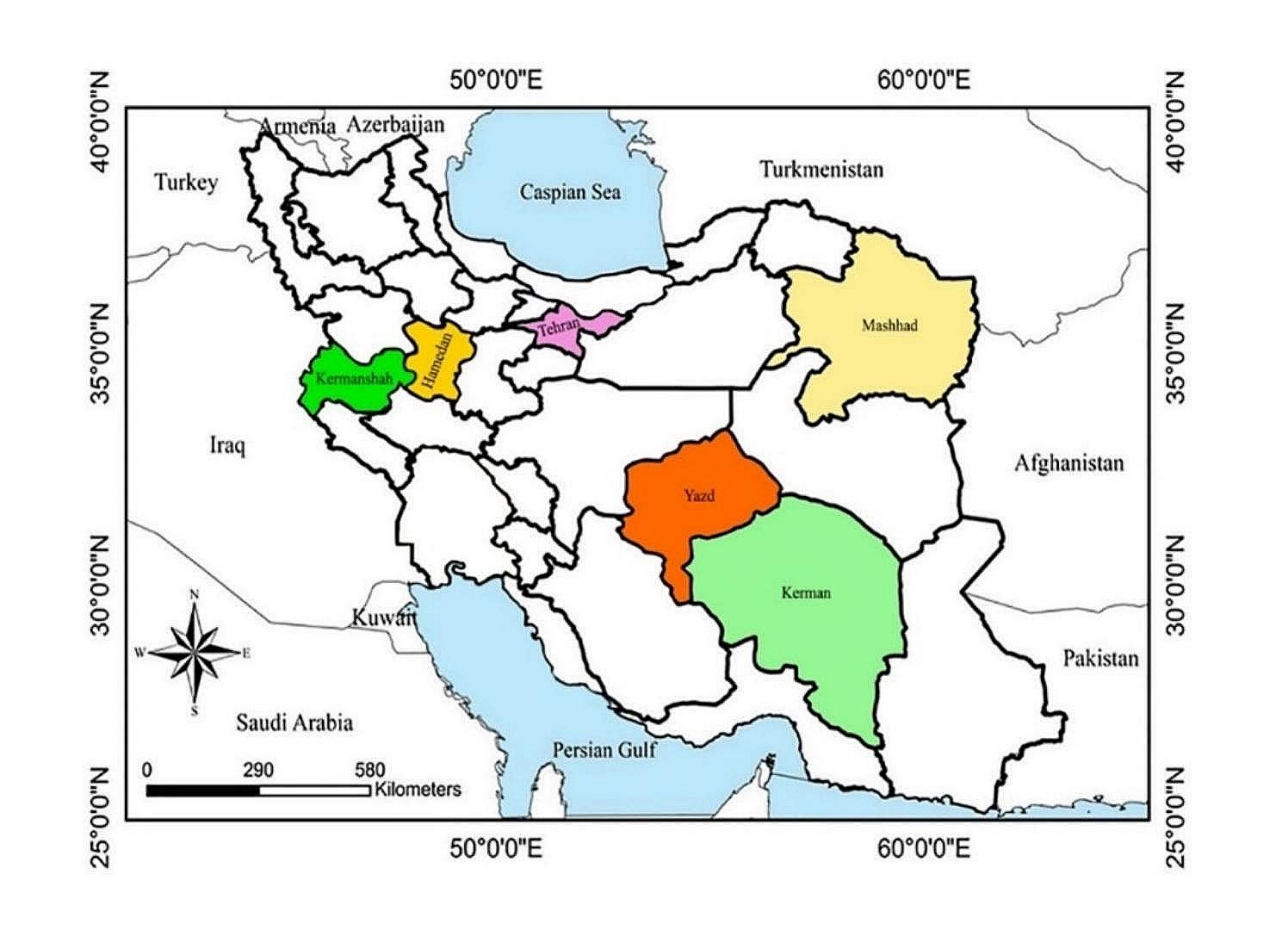
Samples were collected from six provinces namely Razavi Khorasan, Kermanshah, Hamedan, Kerman, Tehran, and Yazd in Iran. The map was drawn by using ArcGIS software version 10.3 (https://enterprise.arcgis.com/en/portal/)

From December 2018 to February 2023, a total of 361 blood samples were collected from cephalic or saphenous vein of cats residing in six provinces of Iran with different climates namely Khorasan Razavi in the northeastern (*n =* 104; cold semi-arid), Kermanshah in the west (*n* = 85; warm and temperate), in the west Hamedan (*n =* 52; cold semi-arid), Kerman (*n =* 46; cold desert climates) in the southeast, Tehran in the central-north (*n =* 45; cold semi-arid), and Yazd in the center (*n =* 29; hot and arid)(Fig. 3). Blood samples were collected in sterile labeled tubes containing Ethylenediaminetetraacetic acid (EDTA) anticoagulant and stored at -70 ºC until DNA extraction. Animal data obtained at the time of sampling included gender, breed, age, and living conditions (Table 1).

### Genomic DNA extraction and PCR amplification

Genomic DNA was extracted from 200 µL of the blood samples using FavorPrep™ Blood Genomic DNA Extraction Mini Kit (Favorgen, Pingtung, Taiwan) according to the manufacturer’s instruction and stored at − 20 ºC until further analysis.

The conventional PCR (cPCR) assays were performed by universal *Mycoplasma* spp. primers targeting the partial sequence of the 16 S rRNA gene [51]. Subsequently, identification of CMhm, Mhf, and CMt in PCR-positive samples was performed using a panel of three species-specific primers in cPCR [22]. PCR reactions were performed in a 25 µL volume reaction mixture containing 12.5 µL of Taq DNA Polymerase 2X Mastermix (Ampliqon, Odense, Denmark), 2 µl of the template DNA, 1 µL of 10 pmol of each forward and reverse primer (synthesized by metabion international AG, Planegg, Germany), and 8.5 µL distilled deionized water. In each run, positive DNA controls that were kindly provided by Professor Dr. Roberta Iatta (University of Bari, Italy), and distilled deionized water were used as positive and negative controls. PCR amplification were run in a SimpliAmp™ thermal cycler (Thermo Fisher Scientific, Waltham, MA, USA) with conditions as described in Table 2. The PCR amplification products were documented using UV Imager (Transilluminator, Vilber Lourmat, France) after electrophoresis in a 1% agarose gel (SinaClon, Tehran, Iran) at 100 V for 60 min.

**Table 2:** PCR conditions and primers targeting 16 S rRNA gene of hemotropic *Mycoplasma* species in this study

Primer name: 5’→3’ sequence	Specificity	Amplicon size (bp)	Cycling condition
HBT-F: ATACGGCCCATATTCCTACG HBT-R: TGCTCCACCACTTGTTCA	*Mycoplasma* spp.	595	94–10 min; (× 40) 95 °C–30 s, 62 °C–30 s, 72 °C–30 s; 72 °C–10 min
Mhf -F: ATGCCCCTCTGTGGGGGATAGCCG Mhf-R: ATGGTATTGCTCCATCAGACTTTCG	*Mycoplasma haemofelis*	274	94 °C–10 min; (× 35) 94 °C–45 s, 56.8 °C–45 s, 72 °C–45 s; 72 °C–10 min
CMhp-F: CTGGGAAACTAGAGCTTCGCGAGC CMhp-R: ATGGTATTGCTCCATCAGACTTTCG	*Candidatus* M. haemominutum	204	94 °C–10 min; (× 35) 94 °C–45 s, 56.8 °C–45 s, 72 °C–45 s; 72 °C–10 min
CMt-F: AGAGGCGAAGGCGAAAACT CMt-R: CTACAACGCCGAAACACAAA	*Candidatus* M. turicensis	138	94 °C–10 min; (× 35) 95 °C–10 s, 58 °C–30 s, 72 °C–30 s; 72 °C–10 min

### Sequencing and phylogenetic analysis

Six randomly chosen PCR products from all four PCRs were Sanger sequenced bidirectionally using the universal *Mycoplasma* spp. primers (HBT) by an Applied Biosystems 3500 Genetic Analyzer (Thermo Fisher Scientific, MA, USA) in Pishgam Biotech Company (Tehran, Iran). The resulting sequences were edited and trimmed by SnapGene^®^ software (GSL Biotech LLC, Chicago, USA) and compared with deposited data in GenBank^®^ (NCBI) (http://www.ncbi.nlm.nih.gov/genbank) using the BLAST program (http://www.ncbi.nlm.nih.gov/BLAST). Subsequently, the representative sequences generated in this study were submitted to GenBank^®^.

Phylogenetic analysis was performed with Mega X software (Molecular Evolutionary Genetics Analysis version 10) [52], and phylogenetic tree was constructed using the maximum likelihood method [53], including 1000 bootstrap replicates.

### Statistical analysis

Frequency analysis was performed using exact binomial 95% confidence intervals (CIs) for three *Mycoplasma* spp. and cPCR results. Possible associations between hemoplasmosis and potential risk factors including city, gender, breed, age, and living condition were assessed by Fisher’s exact or Chi squared tests using the IBM SPSS Statistics software version 26 programs for Windows (IBM Corp, Armonk, NY). *P* value < 0.05 was considered as statistically significant difference.

## Data Availability

Sequenced data were deposited into the NCBI—GenBank—Nucleotide platform under accession number OQ421226, OQ435906, OQ417618, OQ435905 for *Candidatus* Mycoplasma haemominutum (CMhm), OR581285 for *Mycoplasma haemofelis* (Mhf), and OR581286 for *Candidatus* Mycoplasma turicensis (CMt). and are available at the following URL: (https://www.ncbi.nlm.nih.gov/genbank/).
